# Treatment of Epiphyseal Metastasis to the Proximal Humerus Secondary to Breast Carcinoma: A Case Report

**DOI:** 10.7759/cureus.47564

**Published:** 2023-10-24

**Authors:** Inderpreet Singh, Leighann Krasney, William Civatte, William Parrish

**Affiliations:** 1 Orthopedic Surgery, University of Pittsburgh Medical Center Pinnacle, Harrisburg, USA

**Keywords:** osteosynthesis, proximal humeral metastasis, osteoarticular allograft, shoulder hemiarthroplasty, reconstruction, reverse total shoulder arthroplasty, epiphyseal humeral metastasis, breast carcinoma, metastatic bone disease

## Abstract

Metastasis to the bone is a known risk of breast cancer, with the humerus being the most common upper extremity site of metastases, with most lesions located at the humeral diaphysis. We present a unique case of proximal humeral metastasis involving the epiphysis secondary to primary invasive ductal carcinoma in a middle-aged Caucasian female. It is important to have a high degree of suspicion for metastasis when musculoskeletal pain occurs in breast cancer patients, as it may be masked by common, degenerative conditions about the shoulder girdle. When humeral metastases involve the epiphysis, treatment options are complicated by its location, which jeopardizes the integrity of articular cartilage and the function of the shoulder girdle. External beam irradiation provides pain control in a non-invasive manner, sans surgical risks. Surgical intervention will vary depending on the characteristics of the bony lesion, but the use of endoprosthetics has emerged as the most effective option for restoring range of motion and pain control with acceptable rates of implant survival.

## Introduction

The most common site for metastasis in patients with breast cancer is the bone. In the advanced stages of breast cancer, 65%-75% of patients will develop bone metastases [1-3]. The humerus is the most common upper extremity location and the second most common long bone, after the femur, affected by metastatic disease [1,4]. The overwhelming majority of humeral metastases occur in the proximal two-thirds of the humerus [5]. Cases of metastasis to the humeral epiphysis are not well documented in the current literature, and there are few standardized protocols for the diagnosis and management of these lesions. We present a case of a metastatic lesion to the proximal humeral epiphysis in a patient with a history of invasive ductal carcinoma of the breast. We discuss the details regarding the diagnostic workup and management of this unusual presentation of a breast cancer metastatic bone lesion.

## Case presentation

A 58-year-old female with a history of ovarian cancer was found to have a left lower-outer quadrant breast mass. She was diagnosed with a grade 2 invasive ductal carcinoma (G2 IDC) that was estrogen receptor (ER) and progesterone receptor (PR) positive and human epidermal growth factor (HER2/neu) negative with metastasis to the lymph nodes. She had an extensive family history of breast, colon, prostate, and skin cancer. Upon genetic testing, she had adenomatous polyposis coli (APC)and checkpoint kinase 2 (CHEK2) mutations. She underwent chemotherapy with the AC-T regimen (adriamycin, cyclophosphamide, and paclitaxel) and left breast radiation, followed by tamoxifen. Staging did not reveal any evidence of metastatic disease at presentation.

**Figure 1:**
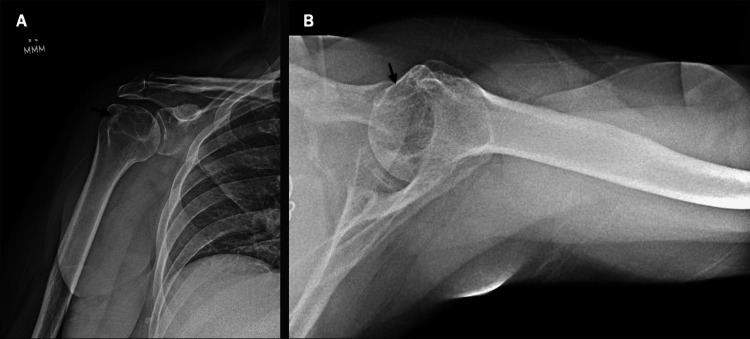
Anteroposterior (A) and axillary lateral (B) radiographs with black arrows are shown in panels A and B, demarcating the large lytic lesion at the superior aspect of the right humeral head adjacent to the greater tuberosity.

Approximately two years later, she developed a traumatic right shoulder pain that radiated down to her wrist. She tried and failed non-operative modalities for her shoulder pain, such as physical therapy and anti-inflammatories. When she presented to orthopedics, her physical exam was concerning for a rotator cuff tear. However, radiographs of the right shoulder demonstrated a large lytic lesion located in the superior aspect of the humeral epiphysis (Figures 1A-1B).

**Figure 2:**
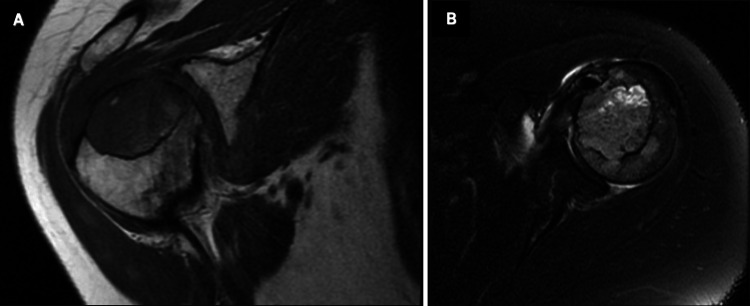
MRI of the right shoulder: coronary T1-weighted (A) and axial T2 fat-suppressed (B) views demonstrating a large humeral head lesion with cortical destruction of the greater tuberosity occupying greater than 75% of the proximal humerus.

Magnetic resonance imaging (MRI) of the shoulder with and without contrast was obtained and demonstrated an aggressive 3.8 x 2.5 x 3.4 cm lesion with adjacent cortical destruction (Figures 2A-2B).

With concern for bony metastasis given her history, she was referred to orthopedic oncology.

**Figure 3:**
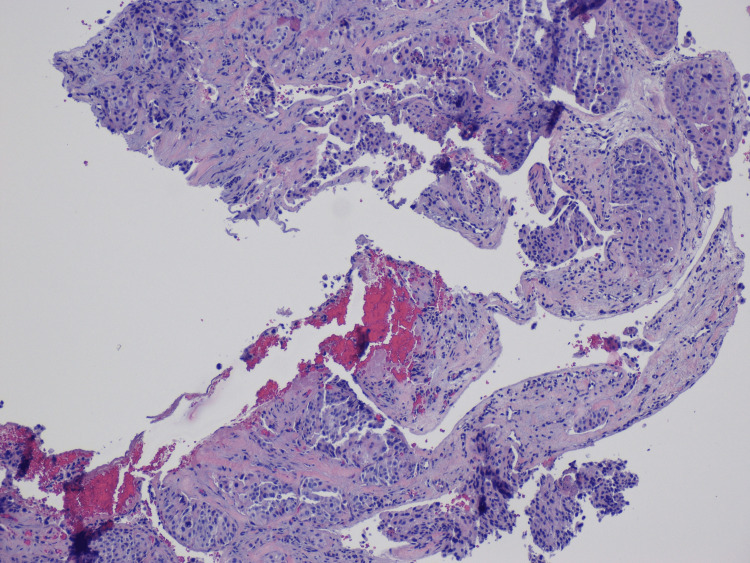
Hematoxylin and eosin (H&E) stain of the specimen from a CT-guided core needle bone biopsy of the right shoulder; immunohistochemical analysis on this biopsy for CAM 5.2 is positive in the malignant cells.

Computed tomography (CT) of the chest, abdomen, and pelvis showed many new pulmonary nodules, a metastatic lesion in the right proximal humerus, and a lytic T12 pedicle lesion. A CT-guided core needle biopsy of the humerus revealed that the lesion was metastatic secondary to breast carcinoma (Figure 3).

Immunohistochemistry demonstrated that this time it was ER/PR/HER2 triple positive. Additionally, hilar lymphadenopathy was noted, and a biopsy demonstrated metastatic disease secondary to recurrent breast carcinoma.

After diagnosis, she was promptly referred to radiation oncology for palliative treatment. Subsequently, external beam radiation therapy was administered as five fractions of 400 cGy. In addition, she received systemic chemotherapy with a regimen of docetaxel, Herceptin, and Perjeta. Several weeks later, her pain was essentially resolved with no discomfort in her shoulder range of motion.

## Discussion

Bone metastasis in breast cancer is a common occurrence, with 65%-75% of advanced breast cancer patients developing metastatic bone lesions [3]. Most metastases are found in the spine, ribs, pelvis, and sternum [6]. Approximately 3.5% of breast cancer patients develop long bone metastases, with 88% of lesions occurring in the femur [7]. When the humerus is involved, almost 90% of lesions are found in the proximal one-third and diaphyseal regions [1]. The main goal of treatment is to improve the patient’s quality of life, as these lesions occur in patients with advanced disease. Consideration between surgical and nonsurgical treatments typically depends on radiographic characteristics, the location of the lesion, and the presence or risk of a pathologic fracture [8].

Currently, there is limited literature on the treatment of epiphyseal lesions and few standardized treatment algorithms to assist in deciding between surgical and nonsurgical management. In our case, the epiphyseal humeral lesion had a cross-sectional width of approximately 75% of the proximal humerus and was associated with cortical bone destruction. Given the cancellous anatomy of the proximal humeral metaepiphysis and the robust rotational forces acting upon it, there is a risk for pathologic fracture [8,9]. Pathologic fractures of the proximal humerus can significantly reduce quality of life as they require a long time to heal, with 50% of cases failing to unite [10]. In addition, the epiphyseal nature of the lesion may make treatment difficult if the patient does not respond to external beam irradiation.

We compared our treatment to an algorithm proposed by Capanna and Campanacci, who defined a classification for surgical indications and methods of treatment for metastasis to the appendicular skeleton, as seen in Table 1 [9].

**Table 1:** Capanna and Campanacci’s classification for treatment of metastatic lesions to the extremities and pelvis Classification described by Capanna and Campanacci [9]

Class	Description
1	Solitary metastatic lesion. Primary with a good prognosis (well-differentiated primary tumors sensitive to adjuvants); Interval >3 years since detection of primary
2	Pathological fracture at any site
3	Impending fracture in a major weight-bearing bone
4	Osteoblastic lesions at all sites. Osteolytic or mixed lesions in non-structural bones (fibula, rib, sternum, clavicle); Osteolytic lesion with no impending fracture in the major weight-bearing bone; Lesions of the wing of the ilium, anterior pelvis, or scapula, excluding class 1 patients

Class 1 through class 3 lesions are typically treated with surgical treatment, while class 4 lesions are initially treated non-operatively. Our patient would fall into the class 3 category given her risk of an impending fracture.

The use of directed external beam radiation therapy along with appropriate chemotherapy is the most common mode of treatment for stable humeral metastases. In general, this will relieve pain and prevent fractures. If breast cancer has spread to multiple skeletal locations, radiation treatment at all sites may not be possible because of limitations in the total amount of radiation that can be given safely. The use of radiotherapy after surgical stabilization shows a significant benefit that improves survival by approximately nine months. Additionally, there is a risk of a loss of fixation in 15% to 20% of patients treated with only surgery without postoperative radiation [8,11]. The choice of surgical treatment does require shared decision-making about whether the short-term morbidity of surgery will outweigh the quality of life improvements. Patients with stable metastatic lesions, or those with limited detriment to function and low impending fracture risk, typically respond well to radiotherapy, especially those secondary to breast carcinoma. This is a desired treatment for most patients, as it limits the morbidity seen with surgical reconstruction and maximizes the improvement in their quality of life.

Reconstruction options are limited for patients with peri-articular involvement of metastatic lesions and a risk of an impending fracture. Osteosynthesis, endoprosthetics, and allograft composites are potential treatment options. The current literature tends to favor endoprosthetics. These can be modular, such as reverse total shoulder modular prostheses, which restore the force couples of the shoulder, allow control of soft tissue tensioning, and re-establish length after resection. Stabilization via surgical intervention should be done in such a manner as to limit the need for multiple procedures.

Hemiarthroplasty (HA) and reverse total shoulder prostheses (rTSA) are the most viable options for a lesion with epiphyseal involvement, but recent studies have favored rTSA. Grosel et al. compared rTSA and HA after resection in patients with primary or metastatic tumors. This study consisted of an older patient population with an average age of 61.8 years for HA and 64.2 years for rTSA. They found improved shoulder range of motion as well as decreased instability and complication rates with rTSA. Patient-reported outcome scores like the American Shoulder and Elbow Surgeons (ASES) Score and the Simple Shoulder Test (SST) were not statistically different. Additionally, recurrence rates in similar studies have been equivocal [12,13].

Alternatively, osteoarticular allografts and allograft-prosthesis composites can be utilized after wide resection for younger patients [4]. Teunis et al. studied the use of these reconstruction options in primary and metastatic lesions. They found similar functional outcomes and implant survival rates among endoprosthesis and allograft-prosthesis composites, whereas osteoarticular allografts were shown to have an increased risk of fracture and lower implant survival [14].

Osteosynthesis with lateral locking plates with or without adjunct poly-methyl-methacrylate (PMMA) bone cement is well studied, with optimal outcomes found in patients with metaphyseal lesions and sufficient epiphyseal bone stock [12,15]. This can be utilized for impending or pathologic fractures of the proximal humerus. Unfortunately, locked plating of metaepiphyseal lesions may fail secondary to insufficient epiphyseal fixation in pathologic bone.

## Conclusions

This case demonstrates the difficulty of managing bony metastases in breast cancer patients. Metastatic disease can mimic musculoskeletal symptoms, and clinicians should have a high index of suspicion for new metastases in patients with a prior history of malignancy, especially breast cancer. Bone metastases to the epiphyseal region of the humerus present a unique challenge. There are non-operative and operative options to treat this type of lesion, and the treatment should be tailored to the needs of each patient.

## References

[1] Aljarrah A, Al-Hashmi M, Malik KA, Sukhpal S, Hussein S, Al-Riyami M, Al-Moundhri M (2013). Mucinous breast cancer with solitary metastasis to humeral head: a case report. Oman Med J.

[2] Pulido C, Vendrell I, Ferreira AR, Casimiro S, Mansinho A, Alho I, Costa L (2017). Bone metastasis risk factors in breast cancer. Ecancermedicalscience.

[3] Macedo F, Ladeira K, Pinho F, Saraiva N, Bonito N, Pinto L, Goncalves F (2017). Bone metastases: an overview. Oncol Rev.

[4] Scotti C, Camnasio F, Peretti GM, Fontana F, Fraschini G (2008). Modular prostheses in the treatment of proximal humerus metastases: review of 40 cases. J Orthop Traumatol.

[5] Thai DM, Kitagawa Y, Choong PF (2006). Outcome of surgical management of bony metastases to the humerus and shoulder girdle: a retrospective analysis of 93 patients. Int Semin Surg Oncol.

[6] Chen WZ, Shen JF, Zhou Y, Chen XY, Liu JM, Liu ZL (2017). Clinical characteristics and risk factors for developing bone metastases in patients with breast cancer. Sci Rep.

[7] Knutson CO, Spratt JS (1970). The natural history and management of mammary cancer metastatic to the femur. Cancer.

[8] Frassica FJ, Frassica DA (2003). Evaluation and treatment of metastases to the humerus. Clin Orthop Relat Res.

[9] Capanna R, Campanacci DA (2001). The treatment of metastases in the appendicular skeleton. J Bone Joint Surg Br.

[10] Clara-Altamirano MA, Garcia-Ortega DY, Martinez-Said H, Caro-Sánchez CHS, Herrera-Gomez A, Cuellar-Hubbe M (2018). Surgical treatment in bone metastases in the appendicular skeleton (Article in Spanish). Rev Esp Cir Ortop Traumatol.

[11] Townsend PW, Rosenthal HG, Smalley SR, Cozad SC, Hassanein RE (1994). Impact of postoperative radiation therapy and other perioperative factors on outcome after orthopedic stabilization of impending or pathologic fractures due to metastatic disease. J Clin Oncol.

[12] Voskuil RT, Mayerson JL, Scharschmidt TJ (2021). Management of metastatic disease of the upper extremity. J Am Acad Orthop Surg.

[13] Grosel TW, Plummer DR, Everhart JS (2019). Reverse total shoulder arthroplasty provides stability and better function than hemiarthroplasty following resection of proximal humerus tumors. J Shoulder Elbow Surg.

[14] Teunis T, Nota SP, Hornicek FJ, Schwab JH, Lozano-Calderón SA (2014). Outcome after reconstruction of the proximal humerus for tumor resection: a systematic review. Clin Orthop Relat Res.

[15] Siegel HJ, Lopez-Ben R, Mann JP, Ponce BA (2010). Pathological fractures of the proximal humerus treated with a proximal humeral locking plate and bone cement. J Bone Joint Surg Br.

